# Additive Manufacturing of Bulk Nanocrystalline FeNdB Based Permanent Magnets

**DOI:** 10.3390/mi12050538

**Published:** 2021-05-10

**Authors:** Dagmar Goll, Felix Trauter, Timo Bernthaler, Jochen Schanz, Harald Riegel, Gerhard Schneider

**Affiliations:** 1 Materials Research Institute, Aalen University, 73430 Aalen, Germany; felix.trauter@hs-aalen.de (F.T.); timo.bernthaler@hs-aalen.de (T.B.); gerhard.schneider@hs-aalen.de (G.S.); 2 Laser Application Center, Aalen University, 73430 Aalen, Germany; jochen.schanz@hs-aalen.de (J.S.); harald.riegel@hs-aalen.de (H.R.)

**Keywords:** additive manufacturing, NdFeB, selective laser melting (SLM), laser powder bed fusion (L-PBF), coercivity, nanocrystalline magnets, permanent magnets

## Abstract

Lab scale additive manufacturing of Fe-Nd-B based powders was performed to realize bulk nanocrystalline Fe-Nd-B based permanent magnets. For fabrication a special inert gas process chamber for laser powder bed fusion was used. Inspired by the nanocrystalline ribbon structures, well-known from melt-spinning, the concept was successfully transferred to the additive manufactured parts. For example, for Nd16.5-Pr1.5-Zr2.6-Ti2.5-Co2.2-Fe65.9-B8.8 (excess rare earth (RE) = Nd, Pr; the amount of additives was chosen following Magnequench (MQ) powder composition) a maximum coercivity of *µ*_0_*H*_c_ = 1.16 T, remanence *J*_r_ = 0.58 T and maximum energy density of (*BH*)_max_ = 62.3 kJ/m^3^ have been achieved. The most important prerequisite to develop nanocrystalline printed parts with good magnetic properties is to enable rapid solidification during selective laser melting. This is made possible by a shallow melt pool during laser melting. Melt pool depths as low as 20 to 40 µm have been achieved. The printed bulk nanocrystalline Fe-Nd-B based permanent magnets have the potential to realize magnets known so far as polymer bonded magnets without polymer.

## 1. Introduction

Additive manufacturing of functional materials offers interesting opportunities: components of complex geometry, efficient use of material and specifically tailored properties. Functional Fe-Nd-B permanent magnet material is important for high-power motor applications. Additive manufacturing of Fe-Nd-B so far mainly focused on processing Magnequench (MQ) powders embedded in polymer using fused deposition modeling (FDM) [1,2,3,4,5] or binder jetting [6]. The fabricated isotropic near-net-shape Fe-Nd-B magnets resulted in magnetic powder isolated and fixed in a polymer matrix (30–50 vol. %). The hard magnetic properties are limited due to the isotropy and the low volume fraction of the hard magnetic phase.

**Figure 1:**
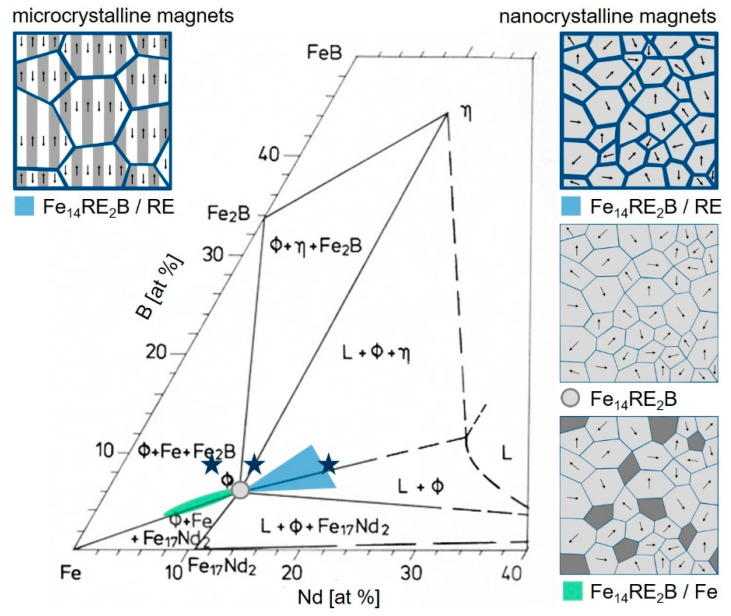
Isothermal section of ternary Fe-Nd-B at *T* = 1000 °C (according to [12]). Schematic microstructures in the demagnetized state are shown for different regions. Compositions addressed in this work are marked in the phase diagram by stars.

Although additive manufacturing based on laser powder bed fusion (L-PBF) is well established to fabricate sintered and fully dense metallic components [7], processing of Fe-Nd-B material by this technique is challenging. There are three reasons for this: First, Fe-Nd-B has an extreme sensitivity to oxidation. Second, there is a lack of powders with a spherical morphology required for a good quality of the printed structures. Third, only quite specific microstructures enable good permanent magnet properties such as high coercivity *H*_c_, high remanence *J*_r_ and high maximum energy density (*BH*)_max_. Such microstructures are composed of small hard magnetic grains on the µm or nm scale (Figure 1). Conventional powder metallurgical processes enable anisotropic microcrystalline magnets with hard magnetic grains of 1–20 µm in size (multi-domain grains) separated by a paramagnetic rare earth (RE) rich grain boundary phase. Conventional melt-spinning results in isotropic nanocrystalline magnets with hard magnetic grains of size 10 to 200 nm (single domain grains). Depending on the choice of the alloy composition three different types of nanocrystalline permanent magnets can be realized. First, magnets with excess RE contain hard magnetic grains which are separated by a paramagnetic RE rich grain boundary phase. Second, stoichiometric magnets are composed of hard magnetic grains which are in direct contact. Third, magnets with overstoichiometric Fe are composites of hard magnetic grains and soft magnetic grains [8,9,10,11].

The only commercially available Fe-Nd-B powder with spherical morphology is MQP-S supplied by Magnequench Corporation (Toronto, ON, Canada). MQP-S powder is an atomized and heat-treated spherical nanocrystalline powder. It shows a mean powder particle size distribution of about 40 μm (*d*_50_: 38 µm, *d*_90_: 65 µm). The flow rate of 3.33 g s^−1^ is comparable to other powders typically used for 3D printing [13]. The nano-sized single-domain FeNdB grains in the powder particles are of uniaxial magnetocrystalline anisotropy. The orientation of the grains is random, so that the bulk magnet shows isotropic magnetic properties [14]. The magnetic properties of the MQP-S powder are coercivity *µ*_0_*H*_c_ = 0.88 T and remanence *J*_r_ = 0.746 T, respectively [14]. MQP-S powder is based on a patented NdPrFeCoTiZrB alloy [15]. Its chemical composition states Nd7.5-Pr0.7-Fe75.4-Co2.5-B8.8-Zr2.6-Ti2.5 (at. %) [13]. It has significantly less amount of rare earth metals (MQP-S 8.2 at. %) compared to powder usually used for the production of conventional sintered magnets (14–18 at. %). The composition prohibits the formation of a Nd rich grain boundary phase. In magnets richer in RE this liquid phase at sintering temperature acts as a magnetically decoupling phase. It is required for realizing large coercivities. However, the coarse and RE-lean powder results in less ignitability and is therefore easier to handle. Research on additive manufacturing of MQP-S powder so far focused on selective laser sintering (SLS) and selective laser melting (SLM). 

Using SLS, the original microstructure and magnetic properties of MQP-S can be retained [14]. Infiltration of low-melting point eutectic alloys into the resulting microporous microstructure after SLS processing led to a Nd-rich grain boundary phase formation and therefore to a substantial enhancement of coercivity by about a factor of two [14]. A similar approach was suggested by Volegov et al. [16]. In a proof-of-concept study they intermixed low melting point eutectic alloy powder with MQP-S powder before SLS processing. In this case, grain boundary infiltration took place in-situ during SLS processing and therefore also led to a significant enhancement of coercivity by a factor of two. The concept so far has been demonstrated for one single layer, but not for bulk.

Using SLM, the original microstructure gets lost when the laser source completely melts the powder material locally during processing. During solidification, soft magnetic Fe is formed due to the over-stoichiometric Fe content. This leads to a significant deterioration of the hard magnetic properties [13] (in the case of laser directed energy process (LMD) a similar behavior has been observed [17]). Recently, it was demonstrated that fine-tuning the laser parameters may result in stabilization of the intended Fe_14_Nd_2_B phase with reduced (but still present) iron segregation for high cooling rates [13,18,19,20]. It turned out that a shallow laser melt pool (melt pool depth <50 µm) in general has to be considered favorable for rapid solidification and coercivities of up to 695 kA/m (0.87 T) [13] 825 kA/m (1.04 T) [18] and 886 kA/m (1.11 T) [19], respectively. A deeper melt pool (depth around 100 µm) led to slow solidification and a coarse microstructure exhibiting poor magnetic properties [13].

Additive manufacturing of non-commercial powder of composition typical for sintered magnets (Fe75-Nd18-B7) was performed by Goll et al. [21,22] using SLM. This allowed to realize very fine microstructures with directed crystal growth and a finely dispersed Nd-rich phase. These model experiments showed possibilities to design and further improve the magnetic properties of sintered Fe-Nd-B magnets. In general, microstructure and related properties of additively manufactured (SLM) components are mainly influenced by processing parameters (laser power, laser scan velocity, scan strategy, hatch distance, substrate conditions, and thickness of powder layer) and powder characteristics (particle shape, rheology, particle size, inner porosity, and purity) for a given chemical composition [23,24,25].

In this paper, a feasibility study is performed to transfer the concept of nanocrystalline Fe-Nd-B magnets from melt-spinning to additive manufacturing technology to achieve permanent magnetic properties in the printed parts. This requires the production of powders of suitable compositions. Furthermore, a special inert gas process chamber for laser powder bed fusion is necessary (including suitable processing parameters) to fabricate the powders into 3D-printed components of the desired microstructures. At the stage of research that is presented, only the optimization of resulting phases, grain size and magnetic properties are investigated. The optimization of process parameters to reduce pores and cracks inside the samples and to achieve a smooth sample surface is ongoing work and will be the topic of forthcoming publications.

## 2. Materials and Methods

For the experiments, three different compositions have been chosen: Nd16.5-Pr1.5-Zr2.6-Ti2.5-Co2.2-Fe65.9-B8.8, Nd11.0-Pr1.0-Zr2.6-Ti2.5-Co2.4-Fe71.6-B8.8 and Nd7.5-Pr0.7-Zr2.6-Ti2.5-Co2.5-Fe75.4-B8.8. The first composition (sample name: P-RE-18) is typical for sintered magnets (excess RE = Nd, Pr). The second composition (P-RE-12) is close to stoichiometry (14:2:1 phase). The third composition (P-RE-8) is exactly the MQP-S composition which is typical for composite magnets (overstoichiometric Fe). Additives such as Pr, Zr, Ti and Co were used in the same proportions as known from MQP-S powder. The compositions of P-RE-12 and P-RE-18 were chosen by adjusting the RE (Nd, Pr) and TM (Fe, Co) content of MQP-S. The content of Zr, Ti and B was kept constant. Production of pre-alloys, preparation of powders and additive manufacturing of components is described in detail in Section 3. Table 1 gives an overview of the samples investigated in this work. 

For the microstructure investigations polished microsections of the samples were produced using metallographic techniques (RotoPol-31, Struers, Willich, Germany). The microstructure was characterized in an optical microscope (Axio Imager.Z2m, ZEISS, Jena, Germany) and in a scanning electron microscope (Sigma 300 VP, ZEISS, Jena, Germany). Scanning electron microscopy including energy dispersive X-ray analysis (EDX) was used to determine the chemical composition of the alloys and occurring phases. Additionally, X-ray diffraction analysis (XRD 3003, CoK_α_ radiation, Bragg Brentano geometry, GE Seifert, Schnaittach-Hormersdorf, Germany) was used to validate the phases present. From magnetometric measurements of the hysteresis loops (PPMS-9T, QuantumDesign, Darmstadt, Germany) the macroscopic magnetic properties (coercivity *H*_c_, remanence *J*_r_, maximum energy density (*BH*)_max_) of the printed magnet parts were determined. For the conversion of the remanence in tesla, a material density of 7.61 g/cm^3^ (Fe_14_Nd_2_B) was used.

## 3. Preparation of Powders and Additive Manufacturing of Parts

### 3.1. Preparation of Powders

**Figure 2:**
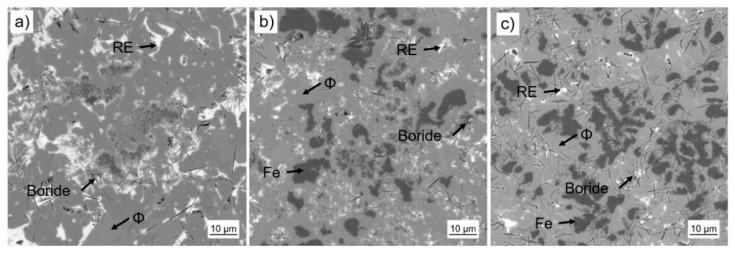
The different Fe-Nd-B based alloys in as cast state (scanning electron microscopy images, backscatter electron detector): (**a**) P-RE-18, (**b**) P-RE-12 and (**c**) P-RE-8. Corresponding phases were assigned to the measured compositions.

**Figure 3:**
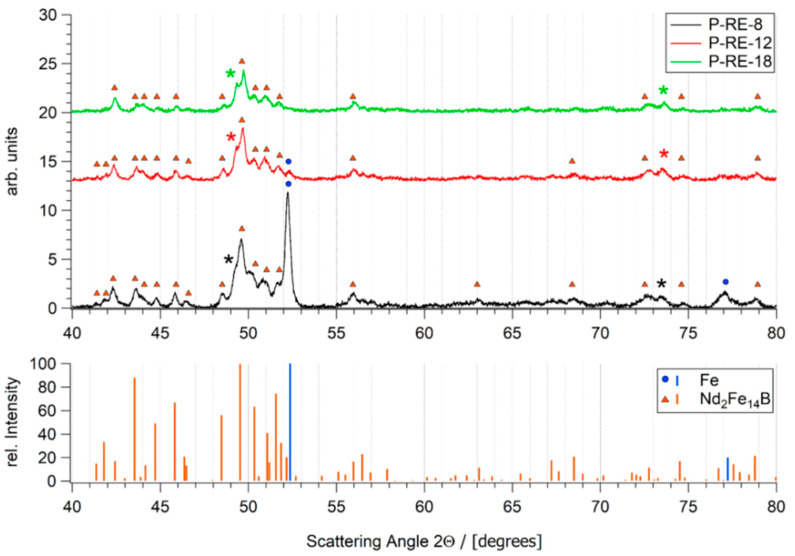
Diffractograms of the three pre-alloys (measured as powders). Detected phases α-Fe and 14:2:1 (ϕ) with their corresponding peaks are shown below. Additionally, the phases that have been identified in the X-ray diffraction (XRD) chart (ϕ phase, α-Fe for P-RE-8 and P-RE-12, ϕ phase for P-RE-18) are marked in the diffractograms. The asterisk marks two unidentified peaks that probably belong to the rare earth (RE) rich phase.

Book-mold-cast pre-alloys of the three different compositions were produced by induction melting (VTC 200 V/Ti, Indutherm, Walzbachtal, Germany) under Ar atmosphere from the constituent elements of high purity (>99.9%) and a Fe-B pre-alloy. SEM images taken with a back scatter detector are shown in Figure 2. The base alloy P-RE-18 shows three main phases: the hard magnetic ϕ phase (14:2:1), the Nd-rich eutectic phase and a fine dispersed boride phase. According to their reduced RE-content, the as cast samples of alloys P-RE-12 and P-RE-8 exhibit an additional α-Fe phase with darker contrast. Additionally, the presence of η phase could be verified via optical microscopy. This phase is not visible in the scanning electron microscopy images because of its atomic density close to that of ϕ phase. The phases observed in the samples were validated using EDX analysis and are in accordance with the phases expected from the phase diagram. The results of the EDX compositional analysis is provided as Supplementary Data (Tables S1–S3). By X-ray diffraction the phases could be verified (Figure 3). For the samples P-RE-8 and P-RE-12, the hard magnetic ϕ phase (14:2:1) and α-Fe phase were identified in the diffractogram. For P-RE-18 only the ϕ phase could be distinguished. In all the measurements two unidentified peaks remain. Those probably belong to the RE rich phase, which can vary in composition and is therefore hard to identify in the measurement. The boride and η phases could not be identified in the measurement because of their low volume fractions.

**Figure 4:**
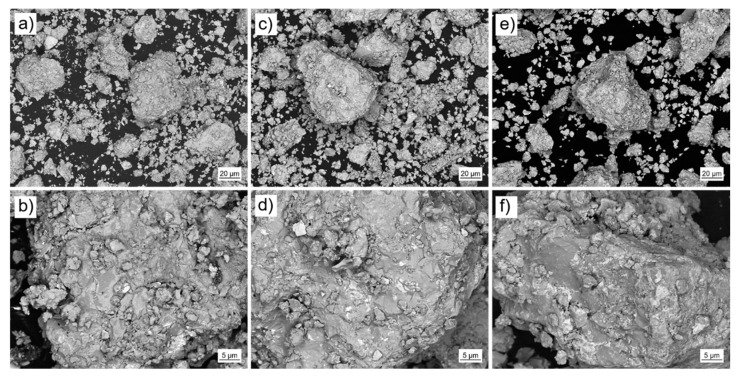
The different Fe-Nd-B based powders used in the laser powder bed fusion (L-PBF) process (scanning electron microscopy images, backscatter electron detector, in lower (top) and higher (bottom) resolution): (**a**,**b**) P-RE-8, (**c**,**d**) P-RE-12 and (**e**,**f**) P-RE-18.

**Figure 5:**
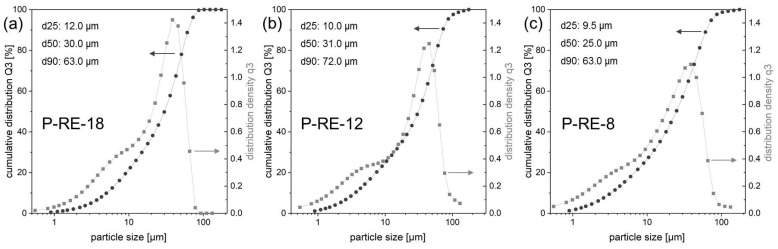
Particle size distribution (cumulative distribution *Q3*, distribution density *q3*) using laser diffraction of the powder fraction < 63 µm for the powder of the alloy (**a**) P-RE-18, (**b**) P-RE-12 and (**c**) P-RE-8. The values of *d25*, *d50* and *d90* are additionally listed.

The pre-alloys were mechanically crushed and ball-milled (Pulverisette 0, Vibratory Micro Mill, Fritsch, Idar-Oberstein, Germany) under Ar atmosphere (O_2_ < 1 ppm). Subsequently, the powder was mechanically sieved (Pulverisette 0, Vibratory Sieve Shaker, Fritsch, Idar-Oberstein, Germany). For additive manufacturing the powder fraction <63 µm was selected (Figure 4). The particle size distribution of the selected fraction was analyzed using laser diffraction (HELOS BR, Sympatec, Clausthal-Zellerfeld, Germany). Figure 5 shows the results for the three different powders (P-RE-18, P-RE-12, P-RE-8). For the measurements a lens system with a measuring range from 0.5 to 175 µm was used. The results are similar for the three different powders. The measured particle distribution shows a distinct peak around 50 µm particle size, a *d90* value of 63.0 µm (P-RE-18, P-RE-8) and of 72 µm (P-RE-12) as well as a *d25* value of around 10 µm. The value *d50* is found in between 25 and 31 µm. Although the aspherical shape of the powder particles is unfavorable, the coating works well in the process chamber. Also, this kind of particle size distribution leads to a high powder density in the powder bed.

### 3.2. Additive Manufacturing of Parts Using Laser Powder Bed Fusion (L-PBF) 

**Figure 6:**
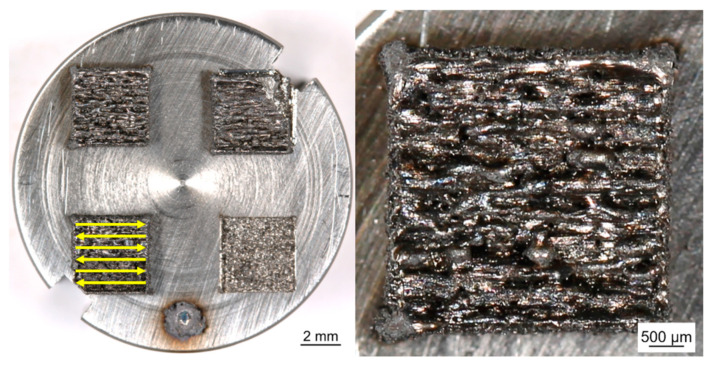
Example of printed Fe-Nd-B selective laser melting (SLM) part (P-RE-18) in top view. Scan strategy is illustrated by yellow arrows.

For lab scale L-PBF of the samples a specific process chamber was developed. The process chamber enables manual scraper operation and processing of small powder volumes (<350 mm^3^). It can be loaded and operated under a very pure Ar atmosphere (O_2_ < 20 ppm). For the experiments the chamber was connected to a fiber laser (TruFiber 1000 with a maximum output power of 1000 W, TRUMPF, Ditzingen, Germany). Samples were built on a substrate plate in layers from the different powder materials. The powder layers were locally consolidated by melting using the laser beam. The size of the printed cuboids was (4 × 4 × 2) mm^3^. Processing parameters were laser scanning speed 2000 mm/s, laser spot diameter 46 µm, hatch distance 30 µm, layer thickness 50 µm and laser power 200 W. The scanning strategy was realized in parallel lines of alternating direction (forward-backwards) (see Figure 6). The laser parameters were determined from preliminary trials so that the laser melt solidifies as quickly as possible and, therefore, achieves a nanocrystalline microstructure with as small as possible grains. Other sample characteristics like cracks, porosity or surface roughness were not considered in this stage of research.

## 4. Analysis of L-PBF Printed Parts

### 4.1. L-PBF of Fe-Nd-B Based Material with Excess RE

**Figure 7:**
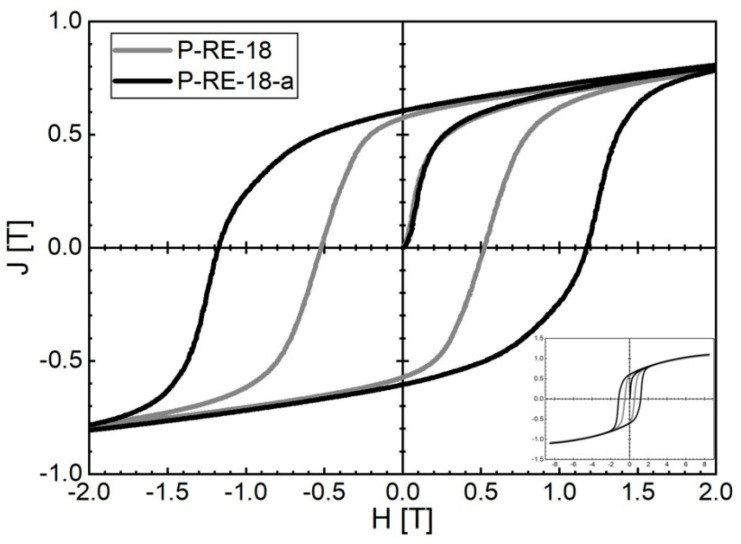
Room temperature hysteresis loops of P-RE-18 produced by L-PBF additive manufacturing: as-built state (P-RE-18, dark grey) and state after two-step post-annealing (600 °C for 10 min and 500 °C for 60 min) (P-RE-18-a, black).

L-PBF of P-RE-18 already leads to good hard magnetic properties for the as-built state. Magnetic properties of *µ*_0_*H*_c_ = 0.52 T, *J*_r_ = 0.57 T and (*BH*)_max_ = 48.0 kJ/m^3^ at room temperature were obtained for the coercivity, remanence and maximum energy density, respectively. The corresponding hysteresis loop is represented in Figure 7. By using a two-step post-annealing treatment similar to sintered magnets (600 °C for 10 min and 500 °C for 60 min) the magnetic properties are significantly improved. Magnetic properties of *µ*_0_*H*_c_ = 1.16 T, *J*_r_ = 0.58 T and (*BH*)_max_ = 62.3 kJ/m^3^ were obtained at room temperature after post-annealing. The corresponding hysteresis loop is also represented in Figure 7.

**Figure 8:**
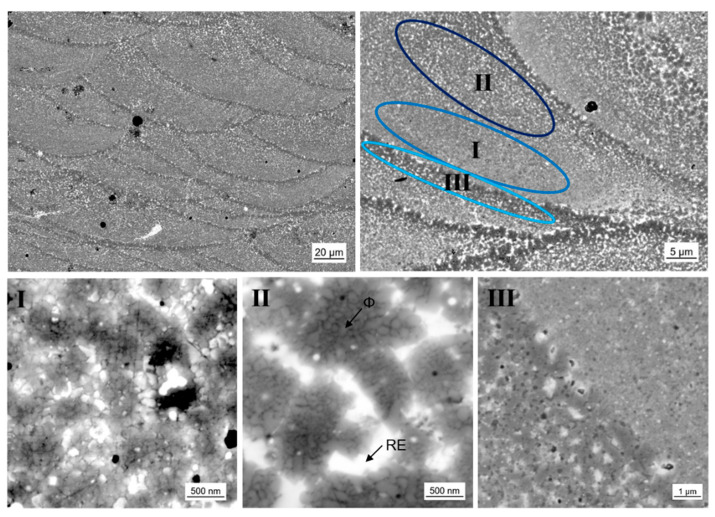
Scanning electron microscopy images using backscatter electron detector of the microstructure of L-PBF sample with excess Nd composition (P-RE-18) using different resolutions. Three different zones occur in/at the laser melt pool, which are highlighted as I, II and III, respectively. The bright regions are assumed to be the RE rich phase, the gray regions/grains to be the hard magnetic ϕ phase and the few black spots to be Fe grains or pores.

The existence of laser melt pools is significant for samples produced by laser additive manufacturing. The melt pool geometry is mainly determined by the laser power and laser scan velocity. The geometry of the melt pool itself and the volume of molten material accordingly influence the solidification behavior and therefore the resulting microstructure. As mentioned above, a shallow melt-pool is considered favorable for rapid solidification. This is needed to develop nanocrystalline material with good magnetic properties. For a high laser scan velocity of *v*_s_ = 2.000 mm/s and a moderate laser power of *P*_L_ = 200 W we achieved samples with a melt pool depth of 20 to 40 µm. In Figure 8 the laser melt pool structure is shown in different magnification. For P-RE-18 three different zones I, II and III with characteristic microstructures can be observed. Zone I occurs mainly in the lower region of the laser melt pool. It consists of nanocrystalline grains with quite narrow grain size distribution and grain sizes of approximately 100 nm. A small amount of a phase with brighter contrast is found between the grains. The phase of brighter contrast is assumed to be the RE rich phase, the grains are assumed to be ϕ phase (the presence of hard magnetic ϕ grains is essential to achieve the magnetic properties shown in Figure 7). The phases observed in the microstructure could not be verified using EDX analysis as they are smaller than the possible lateral resolution. Meaningful indicators for phase allocation, however, are thermomagnetic analysis (measurement of Curie temperature T_C_ of the ϕ phase), XRD analysis, phase diagram information (only a limited number of phases can form from the melt [12,26,27]) and the contrast in backscatter electron mode of the SEM (bright contrast indicates the RE rich phase for the given composition). The XRD analysis and thermomagnetic analysis of the 3D-printed parts are given in the supplementary document (Figures S1 and S2, respectively). Zone II exists in the upper region of the laser melt pool. The zone shows a different microstructure. Larger formations of about 1 to 2 µm in diameter are found. They are surrounded by a phase of bright contrast. The interior of the larger formations consists of nanocrystalline grains. In analogy to zone I, we assume the bright phase to be RE rich and the nanocrystalline phase to be ϕ phase. The size of those nanograins amounts to 80 to 200 nm. Zone III represents the interface of the laser melt pools. In this zone the microstructure is coarsened. Larger grains of size 1 to 2 µm are present, as well as more extended bright regions. This zone belongs to the already solidified material of the laser melt pool below. The energy input of the next layer affects grain growth in this heat-affected zone. The existence of such a heat-affected zone beneath the actual laser melt pool is in accordance with [28] where the change in microstructure after laser spot welding of a sintered magnet is investigated. Although the authors did not report growth of the hard magnetic ϕ grains in the heat-affected zone in the microcrystalline sintered magnet, the nanocrystalline nature of the additively manufactured samples is much more prone to grain growth [29].

### 4.2. L-PBF of Near-Stoichiometric Fe-Nd-B and Fe-Nd-B with Overstoichimetric Fe

**Figure 9:**
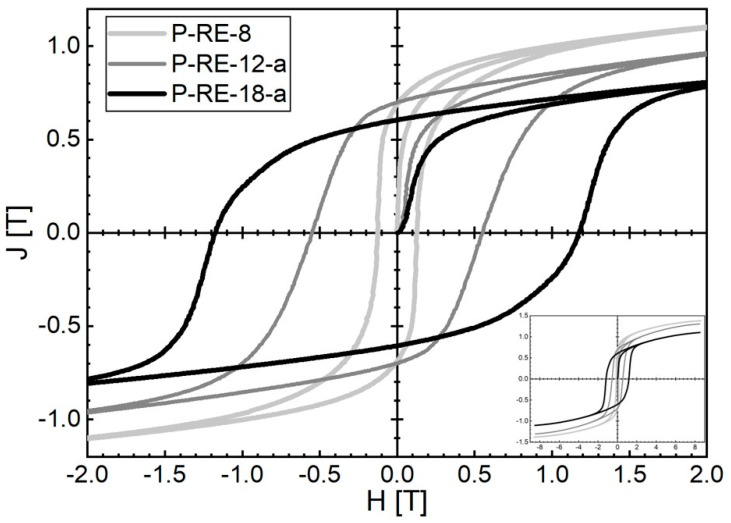
Room temperature hysteresis loops of P-RE-12-a (optimized by post-annealing) (dark grey) and P-RE-8 (light grey) samples produced by L-PBF additive manufacturing. For comparison, the hysteresis loop of P-RE-18-a (optimized by post-annealing) (black) is also shown. It must be noted that the samples with the best magnetic properties achieved so far have been selected in the plot for the three different compositions to demonstrate the potential of the method for the three different regions in the phase diagram to produce bulk nanocrystalline FeNdB.

L-PBF additive manufacturing of P-RE-12 (close-to stoichiometry) results in magnetic properties of *µ*_0_*H*_c_ = 0.55 T, *J*_r_ = 0.70 T and (*BH*)_max_ = 68.1 kJ/m^3^ at room temperature, respectively. Additionally, for this sample the post-annealing treatment (600 °C for 10 min) has been applied to improve the magnetic properties. L-PBF additive manufacturing of P-RE-8 (overstoichiometric Fe) leads to *µ*_0_*H*_c_ = 0.125 T, *J*_r_ = 0.69 T and (*BH*)_max_ = 30.4 kJ/m^3^ at room temperature. For this sample the magnetic properties could not be improved by post-annealing. The corresponding hysteresis loops are also represented in Figure 9.

**Figure 10:**
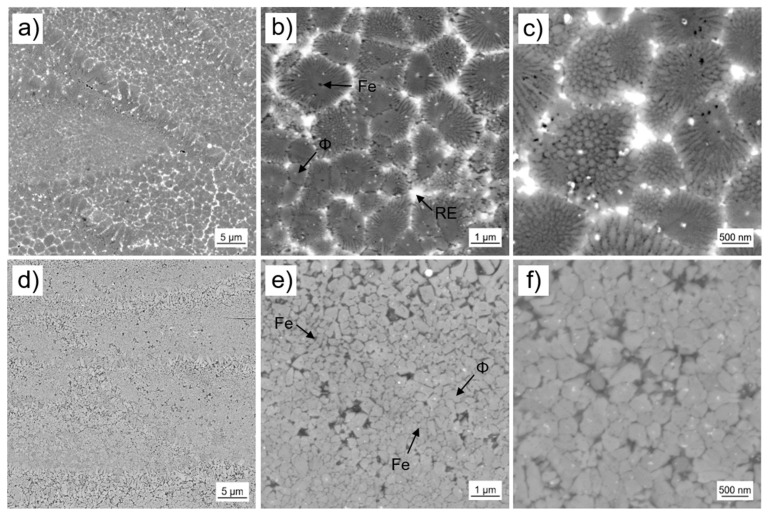
Scanning electron microscopy images using backscatter electron detector of the microstructure of L-PBF samples of close-to stoichiometry composition (P-RE-12) (**a**–**c**) and of overstoichiometric Fe composition (P-RE-8) (**d**–**f**). The bright regions are assumed to be the RE rich phase, the gray regions/grains to be the hard magnetic ϕ phase and the dark spots to be Fe grains.

In contrast to the samples with overstoichiometric Nd (P-RE-18), for the close-to stoichiometry sample (P-RE-12) and the sample with overstoichiometric Fe (P-RE-8) the microstructure inside the laser melt pools is rather consistent throughout the volume (Figure 10). In the case of P-RE-12 (Figure 10a–c) we propose a microstructure that is similar to zone II of P-RE-18 shown in Figure 8. Assumingly, Fe has been primarily formed (black dots in the center of the grains). The hard magnetic ϕ phase formed surrounding the Fe as grains. These ϕ grains are surrounded by structures composed of nanocrystalline grains which could be eutectic. The size of these structures is in the range of 1 to 2 µm, while the grain size of the nanocrystalline grains inside the structures amounts to about 100 to 200 nm. The peritectic solidification of the ϕ phase and eutectic of Fe or η stands in accordance with literature information of the ternary phase diagram Fe-Nd-B [12,30]. A phase of bright contrast (probably Nd-rich ternary eutectic) separates the structures. Similar solidification mechanisms could lead to the nanograin structures in P-RE-18 as shown in Figure 8, zone II. In the case of P-RE-8 (Figure 10e–f) the microstructure inside the laser melt pools consists of grains that are assumed to be of hard magnetic ϕ phase and small grains of darker contrast, that are assumed to be of soft magnetic Fe phase. The grain size distribution of the ϕ grains with grain diameters in between 150 and 500 nm is rather broad. The Fe grains seem to be significantly smaller. For phase allocation the same meaningful indicators have been used as in Section 4.1. The XRD analysis and thermomagnetic analysis of the 3D-printed parts are again given in the supplementary document (Figures S1 and S2). Concerning the heat-affected zone at the laser melt pool interface (region III in Figure 8), both P-RE-12 and P-RE-8 show a thin layer composed of coarser grains (layer thickness: 2–3 µm, grain size: ~1 µm).

## 5. Discussion

### 5.1. Comparison between Nanocrystalline Fe-Nd-B Based Printed Parts and Melt-Spun Material

For melt-spun material it is well-known [8,11]: In decoupled magnets (high coercivity magnets) the hard magnetic Fe_14_Nd_2_B grains are separated by a paramagnetic Nd-rich intergranular film. As the easy axes of the grains are isotropically distributed, the remanence *J*_r_ is limited to *J*_r_ ≤ 0.5 *J*_s_ (*J*_s_: saturation polarization). The coercivity *H*_c_ is rather large (*µ*_0_*H*_c_ > 1.5 T) as each grain behaves like an elementary permanent magnet, that is, for demagnetization the reversed applied field has to overcome the crystal field. Post-annealing at elevated temperatures may further improve the magnetic properties due to an improvement of the surfaces of the ϕ grains (below the melting point of the eutectic of 650 °C a better wetting of the grains can be excluded). In stoichiometric magnets (high coercivity/high remanence magnets) the hard magnetic Fe_14_Nd_2_B grains are in direct contact. They are therefore magnetically coupled by exchange interaction. This results in a magnetic texture at the grain boundaries on the scale of the Bloch wall width (*δ*_B_ = π (*A*/*K*_1_)^0.5^ = 4 nm, *A*: exchange constant, *K*_1_: anisotropy constant). The magnetic texture may lead to an enhancement of the remanence compared to decoupled magnets, at least for grain sizes smaller than 50–100 nm. The exchange coupling between the grains is also the reason for the smaller coercivities compared to decoupled magnets: as part of the magnetic moments is already rotated out of the easy axis, for demagnetization a smaller reversed applied magnetic field is sufficient. In composite magnets (high remanence magnets) nanocrystalline soft magnetic α-Fe grains occur beside the hard magnetic Fe_14_Nd_2_B grains. This may result in a further increase of the remanence. The remanence enhancement is due to the exchange-coupling among the grains as well as to the large spontaneous polarization of α-Fe (*J*_s_ = 2.15 T) which intensifies the magnetic texturing effect. As long as the grain size of the soft magnetic grains is of similar magnitude as the Bloch wall width *δ*_B_ of the hard magnetic grains (<20 nm), the α-Fe grains are fully coupled to the hard magnetic grains. Due to this exchange hardening the soft magnetic properties are not visible macroscopically. With increasing α-Fe content, not only an enhancement of the remanence is achieved, but also a decrease of the coercivity.

For the nanocrystalline Fe-Nd-B based printed parts in this paper the situation seems to be quite similar. With decreasing RE content, the coercivity decreases and the remanence increases continuously. From the feasibility study therefore it can be concluded that the concept of nanocrystalline Fe-Nd-B magnets can be transferred from melt-spinning to additive manufacturing technology to achieve permanent magnetic properties in the printed parts. Novel nanocrystalline structures can be observed. Such structures are larger (spherical) formations composed of 14:2:1 nanograins isolated by an RE rich phase at the boundaries of the clusters. Formation of such novel structures could take place at temperatures between the solidification temperatures of the ϕ phase and the ternary RE-rich eutectic phase. Additionally, the nanograin character for these novel structures is still perfectly fulfilled. Otherwise, a two-step demagnetization behavior would be observed at the remanence, which, however, is not the case. The analysis of this novel microstructure is ongoing work. XRD measurements and high resolution electron microscopy will be used to further investigate the composition, structure and thus the origin of this microstructure. 

The only prerequisite for obtaining nanocrystalline Fe-Nd-B based material is rapid solidification of the melt. In the case of melt-spun material, this is realized by rapid quenching the melt on a fast-rotating copper wheel. In the case of the printed parts the geometry of the laser melt pool reflects the quality of the rapid solidification of the melt. It turned out that small laser melt pool depths are needed to develop nanocrystalline material with good magnetic properties. 

### 5.2. Comparison of P-RE-8 with MQP-S Printed Parts

The chemical composition of MQP-S powder was deliberately chosen in this work. This was to demonstrate that MQP-S with its nanograin structure inside µm-sized spherical powder particles is not a prerequisite to obtain magnetic properties in the printed parts. The process parameters of the L-PBF process are normally chosen to fully melt the powder material locally, so that the original microstructure in any case gets lost during additive manufacturing. The subsequent cooling process after liquefaction in general influences the grain size and the composition of the grain boundaries [31]. A direct comparison of the magnetic properties of our P-RE-8 printed sample with MQP-S printed samples in literature [13,18,19] reveals that the remanence is significantly larger for printed P-RE-8 (*J*_r_ = 0.69 T) compared to the printed MQP-S in literature (0.55–0.63 T), whereas the coercivity is significantly smaller for printed P-RE-8 (*µ*_0_*H*_c_ = 0.125 T) compared to the printed MQP-S in literature (factor 5–8). From this point of view, there is still potential to improve the magnetic properties of the printed P-RE-8 parts by optimizing the process parameters, including cooling conditions. Small grain sizes in nanocrystalline magnets in any case are favorable to fully take advantage of the exchange-coupling effect in such nanostructures. However, as no images of the microstructure in sufficient resolution are given in the papers, it remains unclear whether during additive manufacturing the authors were able to achieve a fully molten state for the MQP-S powder or whether parts of the original nanograin structure were still present after printing. In any case, a nanocrystalline structure shall be present in those MQP-S samples, otherwise no permanent magnet properties would be present. 

### 5.3. Potential

Nanocrystalline ribbons produced by melt-spinning are usually pulverized and mixed with epoxy or thermoplast. This is done to realize isotropic polymer bonded magnets by mold pressing or injection molding of any final shape without cost-intensive after-treatments. Due to the polymer content of typically up to 40 percent by volume, the magnetic values are lower than those of the pure magnet material. Typical magnetic properties of polymer bonded components are, for example, coercivities of 500–1000 kA/m (0.6–1.25 T) and remanences of <0.5 T (for isotropic material) depending on the processing parameters and compositions used. As the Fe-Nd-B parts introduced in this work go along without any polymer, printed bulk nanocrystalline Fe-Nd-B based permanent magnets have the potential to realize magnets known so far as polymer bonded magnets without any polymer.

## 6. Conclusions

A feasibility study was successfully performed to demonstrate that the concept of nanocrystalline Fe-Nd-B magnets can be transferred from melt-spinning to additive manufacturing technology to realize bulk nanocrystalline Fe-Nd-B based permanent magnets. For the investigations, pre-alloys on the basis of Nd-Pr-Zr-Ti-Co-Fe-B with varying RE = (Nd, Pr) content as well as powders thereof and L-PBF printed parts thereof were produced and used. For additive manufacturing a special inert gas process chamber was necessary to safely handle the Fe-Nd-B based powders which are extremely sensitive to oxidation. For example, for Nd16.5-Pr1.5-Zr2.6-Ti2.5-Co2.2-Fe65.9-B8.8 (excess rare earth RE; amount of additives was chosen following MQ powder) a maximum coercivity of *µ*_0_*H*_c_ = 1.16 T, remanence *J*_r_ = 0.58 T and maximum energy density of (*BH*)_max_ = 62.3 kJ/m^3^ was achieved. A very important prerequisite for the development of bulk nanocrystalline Fe-Nd-B permanent magnets with good magnetic properties is the practicability of rapid solidification during laser additive manufacturing. The quality of rapid solidification is reflected in the laser melt pool depth which was as low as 20 to 40 µm in the present study. In addition to the classical nanostructures known from melt-spinning novel nanocrystalline structures (such as clusters of 14:2:1 nanograins isolated by RE rich phase at the boundaries of the clusters) were found. The further investigation and optimization of these microstructures with nm sized features is ongoing work. By optimizing the laser parameters, the porosity, fracturing behavior and also the refinement of the microstructure can be further improved. This may also further improve the magnetic properties. In any case, in the future, the printed bulk nanocrystalline Fe-Nd-B based permanent magnets have the potential to realize magnets known so far as polymer bonded magnets without any polymer. 

## Figures and Tables

**Table 1:** Overview of samples nomenclature, chemical composition, sample state (as built or annealed) and magnetic properties coercivity *µ*_0_*H*_C_, remanence *J*_r_ and maximum energy product (*BH*)_max_.

Sample Name	Chemical Composition	State	*µ*_0_*H*_c_ (T)	*J*_r_ (T)	*(BH)*_max_ (kJ/m³)
P-RE-18	Nd16.5-Pr1.5-Zr2.6-Ti2.5-Co2.2-Fe65.9-B8.8	as-built	0.520	0.57	48.0
P-RE-18-a	Nd16.5-Pr1.5-Zr2.6-Ti2.5-Co2.2-Fe65.9-B8.8	annealed; 600 °C, 10 min; 500 °C, 60 min	1.160	0.58	62.3
P-RE-12	Nd11.0-Pr1.0-Zr2.6-Ti2.5-Co2.4-Fe71.6-B8.8	as-built	0.480	0.69	64.3
P-RE-12-a	Nd11.0-Pr1.0-Zr2.6-Ti2.5-Co2.4-Fe71.6-B8.8	annealed; 600 °C, 10 min	0.550	0.70	68.1
P-RE-8	Nd7.5-Pr0.7-Zr2.6-Ti2.5-Co2.5-Fe75.4-B8.8	as-built	0.125	0.69	30.4

## Data Availability

Data sharing not applicable.

## Supplementary Materials

The following are available online at https://www.mdpi.com/article/10.3390/mi12050538/s1, Figure S1: Diffractograms of the three additively manufactured samples P-RE-18-a, P-RE-12-a, P-RE-8., Figure S2: Thermomagnetic measurements of the three additively manufactured samples P-RE-18-a, P-RE-12-a, P-RE-8., Table S1: Results of the EDX compositional analysis: Sample P-RE-18 in the as cast state as presented in Figure 2a, Table S2: Results of the EDX compositional analysis: Sample P-RE-12 in the as cast state as presented in Figure 2b, Table S3: Results of the EDX compositional analysis: Sample P-RE-8 in the as cast state as presented in Figure 2c.

## References

[1] Baldissera A.B., Pavez P., Wendhausen P.A.P., Ahrens C.H., Mascheroni J.M. (2017). Additive manufacturing of bonded Nd–Fe–B—Effect of process parameters on magnetic properties. IEEE Trans. Magn..

[2] Huber C., Abert C., Bruckner F., Groenefeld M., Schuschnigg S., Teliban I., Vogler C., Wautischer G., Windl R., Suess D. (2017). 3D Printing of polymer-bonded rare-earth magnets with a variable magnetic compound fraction for a predefined stray field. Sci. Rep..

[3] Li L., Post B., Kunc V., Elliott A.M., Paranthaman M.P. (2017). Additive manufacturing of near-net-shape bonded magnets: Prospects and challenges. Scr. Mater..

[4] Li L., Jones K., Sales B., Pries J.L., Nlebedim I.C., Jin K., Bei H., Post B.K., Kesler M.S., Rios O. (2018). Fabrication of highly dense isotropic Nd-Fe-B nylon bonded magnets via extrusion-based additive manufacturing. Addit. Manuf..

[5] Huber C., Mitteramskogler G., Goertler M., Teliban I., Groenefeld M., Suess D. (2020). Additive manufactured polymer-bonded isotropic NdFeB magnets by stereolithography and their comparison to fused filament fabricated and selective laser sintered magnets. Materials.

[6] Paranthaman M.P., Shafer C.S., Elliott A.M., Siddel D.H., McGuire M.A., Springfield R.M., Martin J., Fredette R., Ormerod J. (2016). Binder jetting: A novel NdFeB bonded magnet fabrication process. JOM.

[7] Kruth J.P., Froyen L., van Vaerenbergh J., Mercelis P., Rombouts M., Lauwers B. (2004). Selective laser melting of iron-based powder. J. Mater. Process. Technol..

[8] Kronmueller H., Fischer R., Seeger M., Zern A. (1996). Micromagnetism and microstructure of hard magnetic materials. J. Phys. D Appl. Phys..

[9] Goll D., Seeger M., Kronmueller H. (1998). Magnetic and microstructural properties of nanocrystalline exchange-coupled PrFeB permanent magnets. J. Magn. Magn. Mater..

[10] Goll D., Kronmueller H. (2000). High performance permanent magnets. Naturwissenschaften.

[11] Goll D. (2002). Micrmagnetism and microstructure—Tailoring of high-performance permanent magnets. Z. Met..

[12] Schneider G., Henig E.-T., Petzow G., Stadelmaier H. (1986). Phase relations in the system Fe-Nd-B. Z. Met..

[13] Jaćimović J., Binda F., Herrmann L.G., Greuter F., Genta J., Calvo M., Tomše T., Simon R.A. (2017). Net shape 3D printed NdFeB permanent magnet. Adv. Eng. Mater..

[14] Huber C., Sepehri-Amin H., Goertler M., Groenefeld M., Teliban I., Hono K., Suess D. (2019). Coercivity enhancement of selective laser sintered NdFeB magnets by grain boundary infiltration. Acta Mater..

[15] Kaneyiko H., Miyoshi T., Hirosawa S. (2004). Nanocomposite Magnet and Method for Producing Same. U.S. Patent.

[16] Volegov A.S., Andreev S.V., Selezneva N.V., Ryzhikhin I.A., Kudrevatykh N.V., Mädler L., Okulov I.V. (2020). Additive manufacturing of heavy rare earth free high-coercivity permanent magnets. Acta Mater..

[17] Sridharan N., Cakmak E., List F.A., Ucar H., Constantinides S., Babu S.S., McCall S.K., Paranthaman M.P. (2018). Rationalization of solidification mechanism of Nd–Fe–B magnets during laser directed-energy deposition. J. Mater. Sci..

[18] Urban N., Meyer A., Kreitlein S., Leicht F., Franke J. (2017). Efficient near net-shape production of high energy rare earth magnets by laser beam melting. AMM.

[19] Bittner F., Thielsch J., Drossel W.-G. (2020). Laser powder bed fusion of Nd–Fe–B permanent magnets. Prog. Addit. Manuf..

[20] Jacimovic J., Christen T., Dénervaud E. (2020). Self-organized giant magnetic structures via additive manufacturing in NdFeB permanent magnets. Addit. Manuf..

[21] Goll D., Vogelgsang D., Pflanz U., Hohs D., Grubesa T., Schurr J., Bernthaler T., Kolb D., Riegel H., Schneider G. (2019). Refining the microstructure of Fe-Nd-B by selective laser melting. Phys. Status Solidi RRL.

[22] Goll D., Schurr J., Trauter F., Schanz J., Bernthaler T., Riegel H., Schneider G. (2020). Additive manufacturing of soft and hard magnetic materials. Procedia CIRP.

[23] Liao H.-T., Shie J.-R. (2007). Optimization on selective laser sintering of metallic powder via design of experiments method. Rapid Prototyp. J..

[24] Matthews M.J., Guss G., Khairallah S.A., Rubenchik A.M., Depond P.J., King W.E. (2016). Denudation of metal powder layers in laser powder bed fusion processes. Acta Mater..

[25] Rashid R., Masood S.H., Ruan D., Palanisamy S., Rahman Rashid R.A., Brandt M. (2017). Effect of scan strategy on density and metallurgical properties of 17-4PH parts printed by Selective Laser Melting (SLM). J. Mat. Process. Tech..

[26] Matsuura Y., Hirosawa S., Yamamoto H., Fujimura S., Sagawa M., Osamura K. (1985). Phase Diagram of the Nd-Fe-B Ternary System. Jpn. J. Appl. Phys..

[27] Goll D., Schweizer S., Wegierski C., Schneider G. (2012). Towards a better understanding of intergranular phases in Fe-Nd-B sintered magnets. Phys. Status Solidi RRL.

[28] Chang B., Du D., Yi C., Xing B., Li Y. (2016). Influences of laser spot welding on magnetic property of a sintered NdFeB magnet. Metals.

[29] Lian J., Valiev R.Z., Baudelet B. (1995). On the enhanced grain growth in ultrafine grained metals. Acta Metall. Mater..

[30] Henig E.-T., Schneider G., Stadelmaier H.H. (1987). Metastable solidification of Fe-rich iron-neodymium-boron alloys. Z. Met..

[31] Kronmueller H., Durst K.-D., Sagawa M. (1988). Analysis of the magnetic hardening mechanism in REFeB permanent magnets. J. Magn. Magn. Mater..

