# Supplementary material for: Additive Manufacturing of Bulk Nanocrystalline FeNdB Based Permanent Magnets
Source: Micromachines (Basel). 2021 May 10;12(5):538. doi: 10.3390/mi12050538 (PMC8150944; doi:10.3390/mi12050538)
Supplement: Supplementary file 1 [file micromachines-12-00538-s001.zip › micromachines-1192450-supplementary.pdf]

micromachines

MDPI

Supplementary Material S1

EDX compositional analysis

To verify the composition of the as cast samples as well as of the phases, energy dispersive X-ray analysis (EDX) of the compositions was performed. The spectra were acquired at 20 kV acceleration voltage. Boron cannot be quantified using EDX analysis, unless the B content is very high (as it is the case for the Boride phase). To match the measurement results with the phases in the phase diagram, a theoretical composition of those phases without boron can be calculated. For the ϕ phase this leads to a RE content of 11.76 at-%. This can then be compared to the EDX quantification results. In the same manner, the theoretical B content of the alloy compositions of 8.8 at-% has to be taken into account. Because of the similar atomic density, the η phase cannot be distinguished in the SEM images. Therefore it could not be measured using EDX.

Table S1. Results of the EDX compositional analysis: Sample P-RE-18 in the as cast state as presented in Figure 2a.

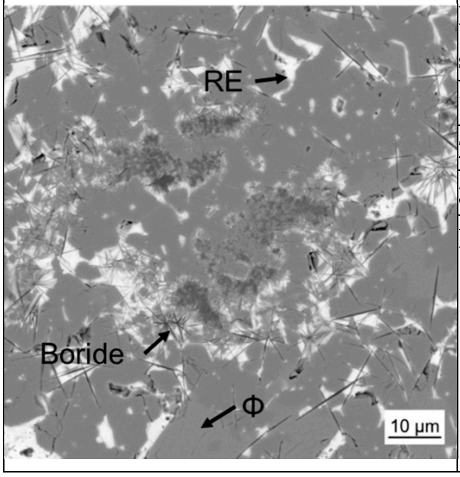

**Measuring spot**	**Nd** [at-%]	**Pr** [at-%]	**Zr** [at-%]	**Ti** [at-%]	**Fe** [at-%]	**Co** [at-%]	**B** [at-%]
1: RE	68.3	8.3	3.6	2.2	12.6	5.0	-
2: ϕ	10.6	0.8	0.4	1.3	84.9	2.0	-
3: Boride	3.1	0.4	9.9	9.2	9.6	0.5	67.3
Full area	18.3	1.8	3.0	2.9	71.4	2.6	-

Table S2. Results of the EDX compositional analysis: Sample P-RE-12 in the as cast state as presented in Figure 2b.

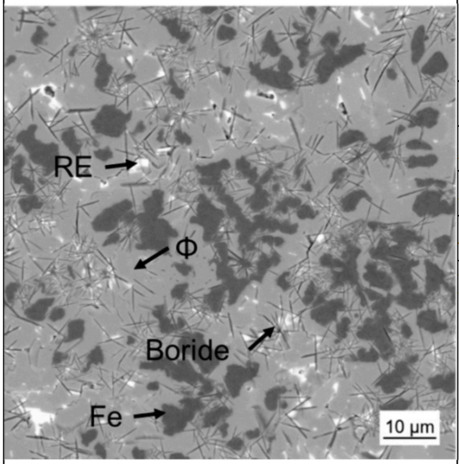

**Measuring spot**	**Nd** [at-%]	**Pr** [at-%]	**Zr** [at-%]	**Ti** [at-%]	**Fe** [at-%]	**Co** [at-%]	**B** [at-%]
1: ϕ	10.1	0.8	4.0	0.8	82.0	2.3	-
2: Fe	0.8	0.1	3.4	1.0	92.9	1.8	-
3: RE	55.0	7.1	6.7	4.1	12.7	14.4	-
4: Boride	2.3	0.3	12.5	13.0	10.1	0.5	61.3
Full area	12.5	1.3	2.7	2.9	77.8	2.8	-

Table S3. Results of the EDX compositional analysis: Sample P-RE-8 in the as cast state as presented in Figure 2c.

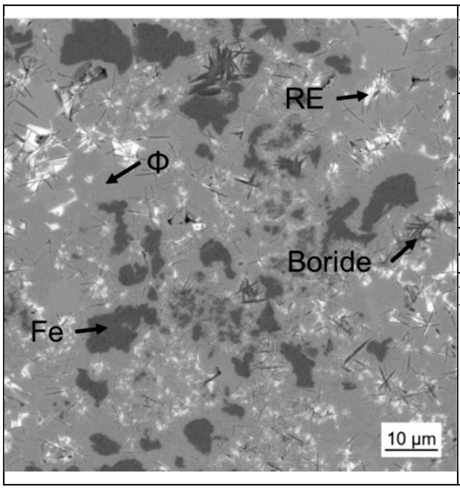

Measuring spot	Nd [at-%]	Pr [at-%]	Zr [at-%]	Ti [at-%]	Fe [at-%]	Co [at-%]	B [at-%]
1: ϕ	0.7	-	-	1.0	96.2	2.1	-
2: Fe	10.5	1.0	0.3	1.0	83.8	3.4	-
3: RE	36.6	5.3	15.9	9.9	22.8	9.5	-
4: Boride	2.2	0.3	10.1	8.2	18.9	0.7	59.6
Full area	8.9	0.9	2.8	2.9	81.7	2.8	-

XRD analysis of 3D printed parts

The presence of ϕ phase in the three different 3D-printed parts was verified by XRD analysis of the 3D-printed parts. For P-RE-8 also the α-Fe phase was verified.

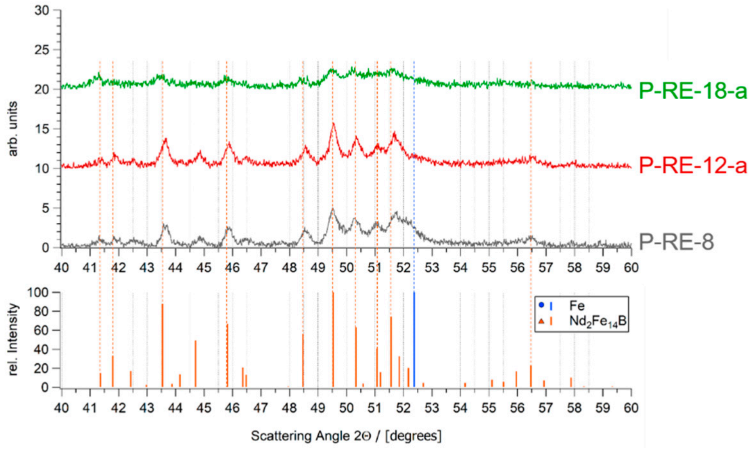

Figure S1. Diffractograms of the three additively manufactured samples P-RE-18-a, P-RE-12-a, P-RE-8. Detected phases α-Fe and 14:2:1 (ϕ) with their corresponding peaks are shown below.

Thermomagnetic analysis of 3D printed parts

To verify the existence of ϕ phase, the Curie temperature has been used. From magnetization versus temperature magnetometry measurements a Curie temperature of T_C = 565 K has been determined, which exclusively fits to the hard magnetic ϕ phase. The obtained values of T_C = 587 K for P-RE-12 and of T_C = 580 K for P-RE-8 again only fit to the ϕ phase. For P-RE-8 there occurs a remaining magnetization in the sample after heating above 585 K. This is due to the existence of a ferromagnetic phase of higher Curie temperature. The only possible ferromagnetic phase showing a Curie temperature higher than 585 K and that can form for the given composition is α-Fe.

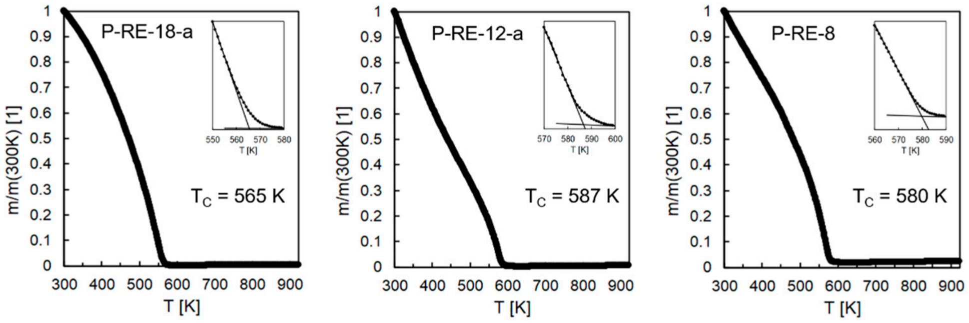

Figure S2. Thermomagnetic measurements of the three additively manufactured samples P-RE-18-a, P-RE-12-a, P-RE-8. Measured Curie temperature T_C of the hard magnetic ϕ phase is noted in the diagrams.
